# Differential strengths of molecular determinants guide environment specific mutational fates

**DOI:** 10.1371/journal.pgen.1007419

**Published:** 2018-05-29

**Authors:** Rohan Dandage, Rajesh Pandey, Gopal Jayaraj, Manish Rai, David Berger, Kausik Chakraborty

**Affiliations:** 1 CSIR- Institute of Genomics and Integrative Biology, New Delhi, India; 2 Academy of Scientific and Innovative Research (AcSIR), New Delhi, India; 3 CSIR Ayurgenomics Unit—TRISUTRA, CSIR- Institute of Genomics and Integrative Biology, New Delhi, India; 4 Department of Ecology and Genetics, Animal Ecology, Evolutionary Biology Centre at Uppsala University, Uppsala, Sweden; Université Paris Descartes, INSERM U1001, FRANCE

## Abstract

Organisms maintain competitive fitness in the face of environmental challenges through molecular evolution. However, it remains largely unknown how different biophysical factors constrain molecular evolution in a given environment. Here, using deep mutational scanning, we quantified empirical fitness of >2000 single site mutants of the Gentamicin-resistant gene (GmR) in *Escherichia coli*, in a representative set of physical (non-native temperatures) and chemical (small molecule supplements) environments. From this, we could infer how different biophysical parameters of the mutations constrain molecular function in different environments. We find ligand binding, and protein stability to be the best predictors of mutants’ fitness, but their relative predictive power differs across environments. While protein folding emerges as the strongest predictor at minimal antibiotic concentration, ligand binding becomes a stronger predictor of mutant fitness at higher concentration. Remarkably, strengths of environment-specific selection pressures were largely predictable from the degree of mutational perturbation of protein folding and ligand binding. By identifying structural constraints that act as determinants of fitness, our study thus provides coarse mechanistic insights into the environment specific accessibility of mutational fates.

## Author summary

Environmental conditions are known to shape natural selection. However, their influence on molecular evolution is still largely unclear. Here, we use a high throughput mutational scanning approach to investigate how representative physical and chemical environments alter mutational fates of an antibiotic resistant gene. From co-culture bulk competitions with purifying selection carried out under different test environments, we obtained empirical fitness of individual single site mutants of the gene. Mutant fitness was found to differ across environments. In order to gain mechanistic insights into the observed environmental influence on mutational effects, we analyzed relative strengths of protein level structural constraints in determining the fitness effects. Remarkably, this analysis revealed a high degree of predictability: overall strengths of environment-specific selection pressures were determined by the degree of mutational perturbation of protein folding and ligand binding. Overall, our results show that these structural constraints act as determinants of environment specific mutational fates.

## Introduction

Environmental conditions shape natural selection and drive rates of organismal adaptation through Genotype-by-Environment Interactions (GEI) and alterations of the genotype-phenotype map linking DNA sequence variation to the expression of quantitative traits [1]. Depending on the environment, such interactions can thus predispose a particular genotype to alternative fates and divergent evolutionary trajectories [2–7]. While the roles of standing variation and de novo mutation in adaptation to new environments have received much theoretical and empirical consideration [8–11], these sources of genetic variation are also likely to differ in fundamental ways. In particular, GEI based on standing variation may differ from GEI from de novo mutation as the former are shaped by selection [12] while the latter will be so to a much lesser extent [13]. Indeed, while the Distribution of Fitness Effects (DFE) of mutations has fundamental consequences for rates of evolution, little is known generally about their environmental specificity [9,14–16].

Chemical and physical properties exercise fundamental constraints on enzymatic reactions and protein function, and in extension, organismal fitness [17]. Thus, in-depth knowledge about environmental influences on biochemical properties and molecular features underpinning phenotypic traits may bring considerable insights and predictive power of organismal adaptation and evolutionary trajectories in heterogeneous and complex environments. Indeed, maintenance of proteostasis is key to survival in stressful environments [18,19] and many diseases are associated with dysfunctional proteostasis machinery [20]. Hence, investigating whether and to what extent proteostasis in terms of intracellular protein folding and stability play a role in determining GEI and environment-specific DFE may be a key step in predicting mutational fates and thereby understanding molecular basis of environmental influences on the genotype-phenotype map.

Monitoring of environment-specific DFEs is greatly enhanced by prospective mutational scanning of single mutants which provide a rapid means to study single steps of molecular evolution, as compared to spontaneous mutations which occur at a very low rate [21]. Deep sequencing based high throughput approaches such as deep mutational scanning [22,23] have now rendered large scale assessment of mutational effects on gene function possible [24]; allowing comprehensive analysis of the sequence-space of a gene. Resultant DFEs of the mutations provide a continuous series of fitness effects ranging from strongly deleterious to beneficial, and represent a valuable resource for quantitative genetic research [25]. In recent years, exploration of environmental influence on the DFE of mutations with large-scale genotype to phenotype data has resulted in the identification of environment-specific mutational effects [16,26]. However, qualitative and quantitative identification of determinants of these mutational fitness effects has been challenging [27,28]. Therefore, mechanistic understanding of GEI and environment-specific DFE is much needed in order to increase the robustness in current approaches of predicting genotype-phenotype relationships [29,30].

In this study, we monitored the fitness landscape of the Gentamicin (Gm) resistant gene—GmR (aminoglycoside 3-N-acetyltransferase (aacC1)) under different sets of physical and chemical environments. We utilized a single site mutation (SSM) library (>2000 mutants) of the gene, heterologously expressed in *E*. *coli*. We acquired relative fitness of single site mutants of GmR, by carrying out co-culture bulk competition assays that select for the gene’s function, under predominantly purifying selection in different environmental conditions. Adopting a deep mutational scanning approach, preferential enrichments of the mutants were monitored via deep sequencing. The physical environments investigated in this study include growth temperatures; lower (30°C) or higher (42°C) than the optimal growth temperature (37°C) of *E*. *coli*. High temperature is known to severely impair protein folding of temperature-sensitive mutants [31], while low temperature has been shown to induce reversible effects on protein folding [32]. Hence, the influence of temperature on the fitness landscape of GmR may allow us to understand how the requirement of proteostasis limits the gene sequence space available for evolution. Among chemical environments, we studied effects of TMAO (Trimethylamine N-oxide) and glycerol, which are known to act as chemical chaperones that may buffer mutational effects by assisting protein folding via alternative mechanisms [33] [34]. Assessment of the role of such solvent-protein interactions in guiding mutational fates is of particular importance, considering that the solvent accessible surface area of proteins are strong predictors of protein evolution rate [35–37].

The assessed mutational effects depended strongly on the acting environmental conditions, a hallmark of mutational GEI. Moreover, molecular constraints such as protein stability and ligand binding were identified to be common across all test environments. The selection pressures imposed by physical and chemical environments, at minimal concentration of antibiotic, were largely mediated via folding constraints, and hence, could be predicted. For instance, elevated temperature imposed stronger purifying selection against mutants whereas chemical chaperones were found to increase mutational robustness, alleviating deleterious fitness effects (buffering effect). Collectively, through mutational scanning of a conditionally essential gene, this study uncovers how environments guide molecular evolution and assigns a central role to underlying molecular constraints in form of protein folding and ligand binding in determining mutational fates in different environments.

## Results

### Deep mutational scanning of GmR

In order to assess survival and competitive fitness of individual single site mutants, we carried out deep mutational scanning [22,23] of GmR, by carrying out co-culture bulk competitions of a single site mutants (SSM) library (see Materials & Methods). Since antibiotic resistance of GmR is dosage dependent (S1A Fig), the strength of purifying selection (i.e. the concentration of Gentamicin (Gm)) in competition assays was optimized at ~4 fold lower than the inhibitory concentration for wild type GmR while still being higher than the inhibitory concentration for the host (*E*. *coli* K-12) alone (S1B Fig). This moderate purifying selection allows detection of a diverse set of mutants rather than only 'quick fix' outcomes that would be detected at stringent purifying selection [38]. If not mentioned otherwise, 12.5 μg/mL of Gm is therefore used in subsequent deep mutational scanning experiments.

**Fig 1:**
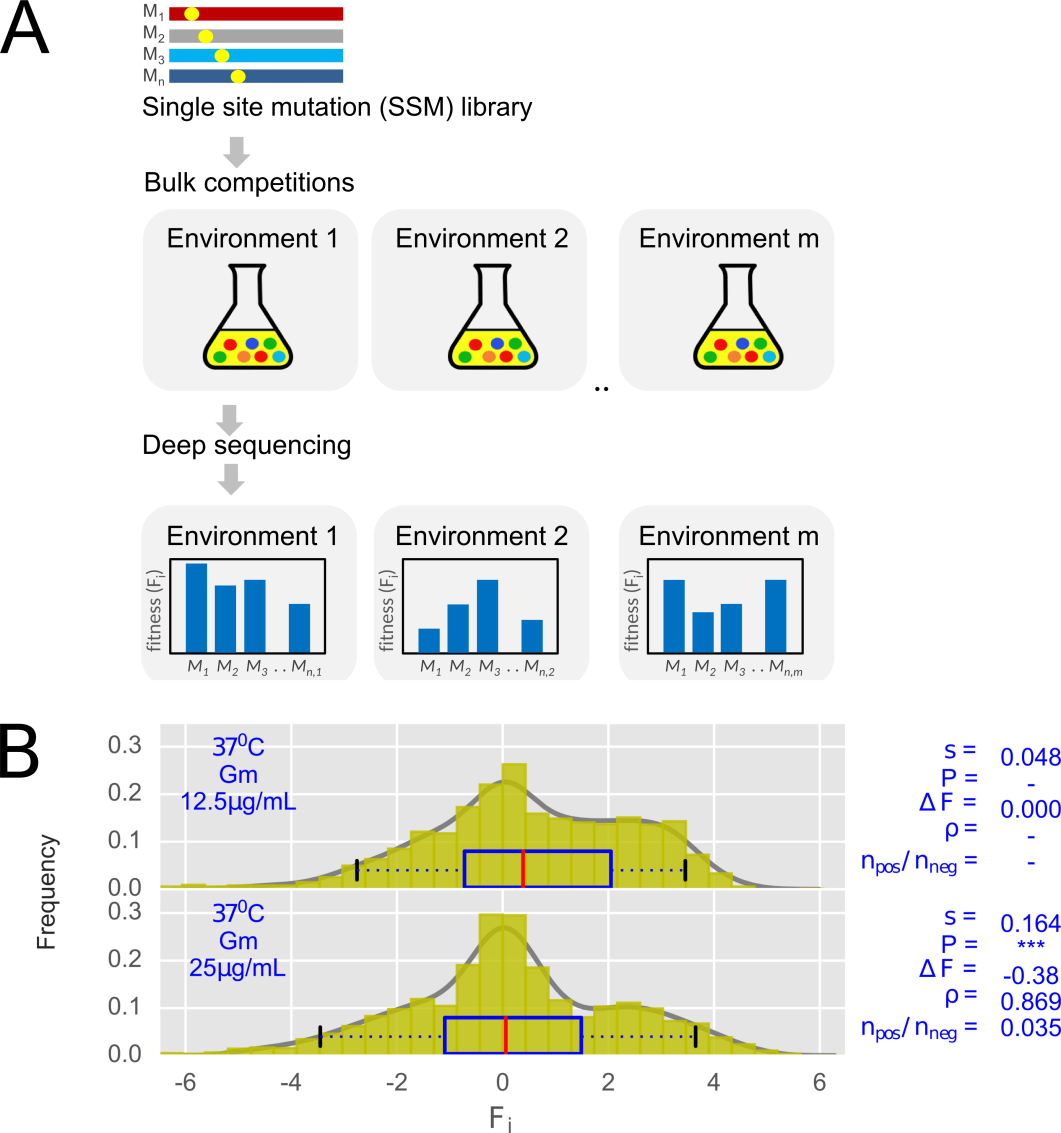
Deep mutational scanning of GmR. (A) Experimental strategy for monitoring survivabilities and competitive fitness of the library of single site mutants of GmR (See Materials & Methods). (B) Comparison between distributions of effect sizes obtained at Gm concentration of 12.5μg/mL (reference) and at 25μg/mL (test). F_i_ denotes fitness score, *s* denotes mean viability selection coefficient. Significant differences between the viability selection coefficient in a specific test environment compared to the reference environment (37°C, 12.5μg/mL) was evaluated by Bayesian MCMC resampling (***, P < 0.001, See Materials & Methods). ΔF is relative change in average fitness. ρ is a mutational robustness score. Distributions are fitted by kernel density estimation. Boxplots show median ± 50 & 95% of the distributions.

For obtaining relative fitness, which would be a proxy for the catalytic activity of the mutants, two parallel co-culture bulk competitions were carried out—one in presence of Gm (selected pool) and another in absence of Gm (unselected pool) (Fig 1A). Optimal growth temperature of *E*. *coli* i.e. 37°C was designated as a reference environment (if not otherwise stated). At the end of bulk competitions, ultra-deep sequencing provided counts of mutants (see Materials & Methods)–that correlated strongly among independent biological replicates (S2 Fig); signifying low inherent noise in the measurements and absence of emergent mutations during the selection process.

Next, relative fitness scores of mutants were calculated by preferential enrichments, i.e. log fold differences between counts of the mutants in the selected pool versus the unselected pool–generating a mutational matrix of fitness effects for each environment (S3 Fig). Note that catalytic fitness scores obtained by this strategy represent maximum asymptotes of mutants’ growth which are different from ‘canonical’ relative fitness estimated from growth rates. Also, completely eliminated highly deleterious mutants were assigned a null fitness. Therefore, unless otherwise mentioned, subsequent analysis of fitness scores is carried out with surviving mutants alone. Upon estimating thresholds for statistically neutral fitness effects (See Materials & Methods, S4 Fig), it was evident that fitness effects of synonymous mutants across all the environmental conditions studied in this work were mostly neutral (S5 Fig). Therefore, subsequent analysis is mainly focused on the fitness effects of non-synonymous mutants.

In order to test whether our experimental system is able to capture the catalytic activities of mutants, we first assessed dosage dependent survival of the mutants. Expectedly, bulk competitions carried out at high dosage of the antibiotic (25 μg/mL Gm) indeed showed a skew towards lower fitness scores (Fig 1B). The fitness effects of mutants in a given environment were captured through following 4 parameters (S1 Table). (1) Mean viability selection coefficient (*s*) against non-synonymous mutations: *s* = 1 –[*v*^non^/*v*^syn^], where, *v*^non^ and *v*^syn^ are mean viabilities of the non-synonymous and synonymous mutants respectively. A higher value of *s* thus indicates decreased relative survival of all non-synonymous mutants in the given environment. (2) Change in average fitness (ΔF) equals F_test_- F_ref_, where, F_test_ and F_ref_ are average fitness of all mutants of a given test environment and that of the corresponding reference environment respectively. A lower value of ΔF would indicate a relative decrease in average fitness. (3) In order to capture mutational robustness in a given environment, a rank correlation coefficient (ρ) between fitness scores of all mutants in a given environment and that in the corresponding reference environment was determined. A high value of ρ indicates higher mutational robustness. Lastly, (4) the ratio of the number of mutants with positive and negative fitness effects (n_pos_/n_neg_) relative to the reference environment is estimated (see Materials & Methods). Among these 4 parameters, the mean viability selection coefficient (*s*) is a direct estimate of the mean strength of selection against non-synonymous mutations for a given environmental condition, while the remaining 3 parameters are estimated relative to the reference environment.

Deleterious fitness effect of high Gm-dosage was well captured through the set of 4 parameters. Firstly, selection coefficient *(s)* showed an increase (*s =* 0.164) compared to the reference concentration of 12.5 μg/mL Gm (*s* = 0.048). In terms of relative parameters, average fitness decreases (ΔF = -0.380), mutational robustness is compromised (ρ = 0.869) and a greater number of mutants cause deleterious fitness effects (n_pos_/n_neg_ = 0.035; See Materials & Methods). This dosage dependent deleterious fitness effect is consistent with previous reports from mutational scanning of other antibiotic resistant genes [39–41]. This dosage dependence taken together with a positive correlation between fitness scores and predicted evolutionary rates per site (S6 Fig) signify that the empirical fitness scores indeed capture catalytic activities of GmR mutants.

### Environmental conditions induce variable fitness effects

Next, we tested the two sets of environmental conditions using our experimental system. Firstly, among physical environments, lower (30°C) and higher (42°C) temperature were found to confer moderate (*s* = 0.103) and considerable (*s* = 0.338) increase in mean viability selection respectively, compared to the reference environment of 37°C (*s* = 0.048). For surviving non-synonymous mutants, strong negative effects at 42°C (n_pos_/n_neg_ = 0.14) can be explained by potentially pronounced protein misfolding at high temperature [42]. Note that here the increase in average fitness of surviving mutants (ΔF = 0.19) at 42°C is due to the complete elimination of highly deleterious mutants.

**Fig 2:**
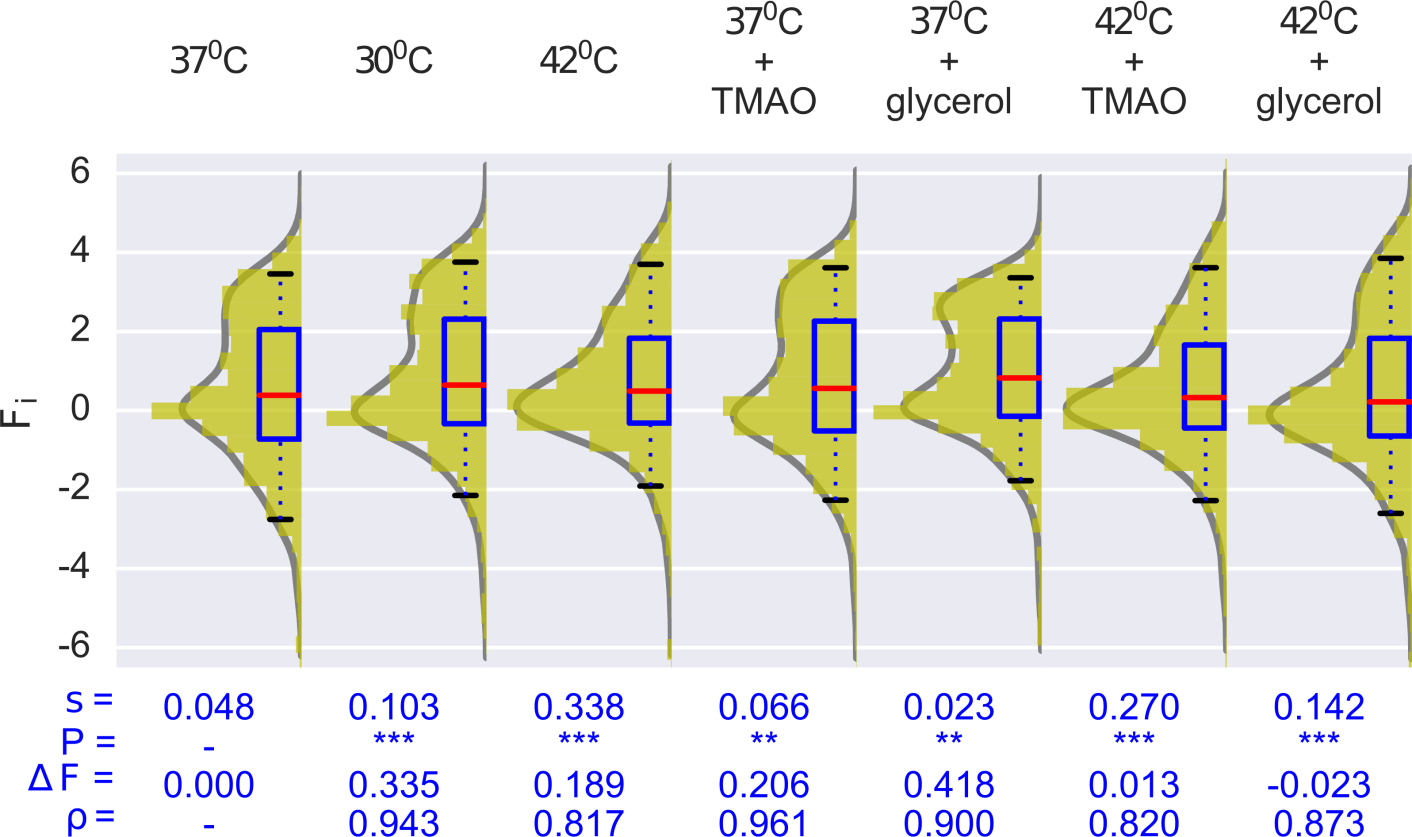
Environmental conditions induce variable fitness effects. Comparative analysis of distributions of effect sizes obtained under various test environments with reference environmental condition i.e. 37°C. F_i_ denotes fitness scores. *s* denotes mean viability selection coefficients against non-synonymous mutants. Significant differences between the viability selection coefficient in a specific test environment compared to the reference environment (37°C, 12.5μg/mL) was evaluated by Bayesian MCMC resampling (**, P < 0.01, *** P < 0.001, See S6 Table and Materials & Methods). ΔF is relative change in average fitness. ρ is the mutational robustness score. Distributions are fitted by kernel density estimation. Boxplots show median ± 50 & 95% of the distributions.

Chemical chaperones–TMAO and glycerol–comprising a set of chemical environments, have relatively weak effects on mean viability selection (*s* = 0.066 and *s* = 0.023 respectively) relative to the reference environment (s = 0.048), with positive fitness effects on growth (n_pos_/n_neg_ = 2.00 and n_pos_/n_neg_ = 33.60 respectively) (Fig 2). Additionally, mutational robustness scores were higher in both the environments (ρ = 0.961 for TMAO and ρ = 0.900 for glycerol) than in the absence of these chemical chaperones. To examine the extent of these positive effects, we analyzed the bulk competitions at high Gm dosage (25 μg/mL) too. There we find that, unlike TMAO (*s* = 0.219), glycerol is still able to provide mutational robustness (*s* = 0.036) (S7 Fig). Collectively, therefore, among the two chemical environments, glycerol seems to exert more pronounced positive effects than TMAO. A possible explanation for this difference may lie in the two chemical chaperones’ alternative mechanisms of aiding protein folding [33].

Having characterized effects of individual environments, we next explored how combinations of environments (complex environments) influenced mutational fitness. Environments with significant and opposing effects on mutational fitness i.e. high temperature in combination with one of the two chemical chaperones–were simultaneously applied in the bulk competitions. There were evident increases in selection relative to the reference environment (37°C: *s* = 0.048) in both cases (*s* = 0.270 and *s* = 0.142 for 42°C + TMAO and 42°C + glycerol, respectively), demonstrating a major effect contributed by high temperature. However, selection was alleviated, and mutational robustness increased, as compared to when high temperature was applied alone (s = 0.338). This demonstrates mutational buffering conferred by the chemical chaperones, which is consistent with an earlier finding [34]. Noticeably, TMAO went from causing a slight increase in the strength of purifying selection at 37°C, to having a buffering effect at 42°C, demonstrating environmental-specificity in the fitness consequences of this chemical chaperone.

### Contextualizing environmental effects in terms of molecular constraints

**Fig 3:**
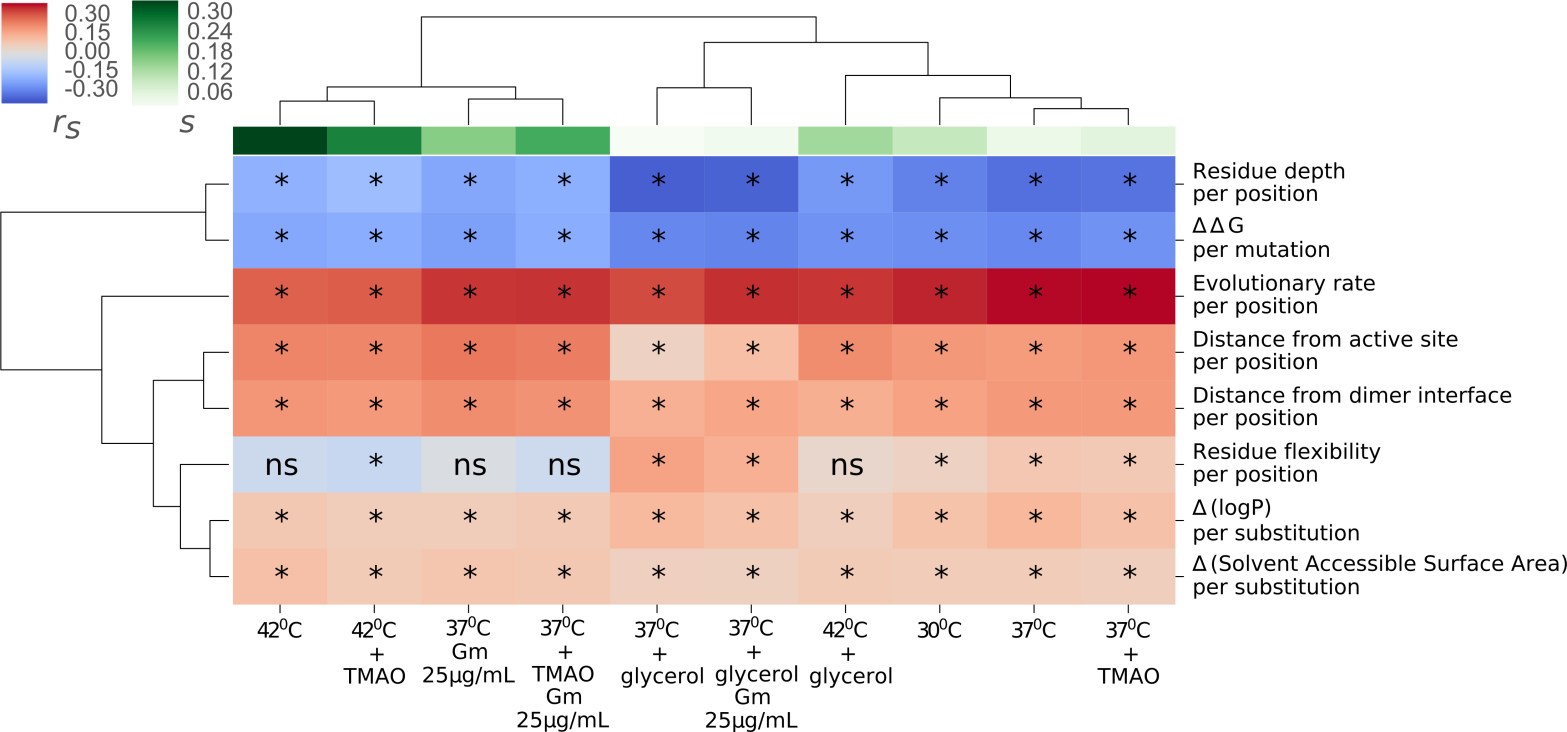
Correlative analysis for identifying environment-specific molecular constraints. A heatmap of Spearman’s rank correlation coefficients for correlations between fitness scores and molecular features (rows) of surviving mutants in each test environment (columns). Each box shows Spearman’s rank correlation coefficient (r_s_) between fitness scores of mutants in an environment (in column) and mutational features (in row). s is mean viability selection coefficient. Euclidean clustering along rows and columns is based on the Spearman’s rank correlation coefficients. *: P < 0.05, ns: non-significant.

In order to gain insights into the mechanistic basis underlying the environmental influence on mutational fitness effects, we scanned a comprehensive set of molecular features of the single site mutations (see Materials & Methods and S2 Data) and correlated these features with the mutants’ fitness score in each of the test environments (Fig 3 and S2 Table). From the Euclidean clustering of these correlation coefficients, it is apparent that the correlations roughly separate the environments with high selection pressure (*s*) from the ones with low selection pressure. This thus suggests that information encoded in the molecular features, to some extent, can predict the selection pressures imposed by each environment.

Among the set of molecular features, evolutionary rate per site (predicted from ConSurf [43]) was found to most strongly correlate with the fitness scores; indicating that even in different environmental conditions, inherent mutational tolerance of a gene is still conserved. However, this feature summarizes individual contributions of various interrelated features. Therefore, in order to gain finer mechanistic understanding, correlations with nearly independent individual structural features are required. Among folding related features– ΔΔG (perturbation of protein stability, predicted from PoPMusic [44]) and residue depth (distance of a residue from the surface of the protein, calculated using MSMS libraries [45]) were negatively correlated with the fitness scores (P<0.0001). Here, residue depth can be considered as a folding feature because mutations at buried sites are known to cause more stability perturbation than mutations at the surface [46]. Effectively, mutations at buried sites of the protein (high ΔΔG) are more likely to be associated with decreased fitness compared to mutations at the surface of the protein (low ΔΔG).

The Distance of mutated residues from active sites of the protein, serving as proxies for potential perturbation of ligand binding, show positive correlations (P<0.05) with fitness scores of surviving mutants across all environments. This suggests that mutations near active sites are more likely to bear fitness costs. Other molecular features were more weakly related to fitness of the surviving mutants in the different environments. Distances of mutation sites from the dimer interface also show positive correlations with fitness scores across all environments, suggesting that dimer formation is an essential condition for proper functionality of the enzyme. In addition, residue flexibility, Δ(logP) per substitution and Δ(Solvent Accessible Surface Area) per substitution were mostly negatively and relatively weakly correlated with the fitness scores. Note that the relatively weak correlations may arise from the combination of uncertainty in estimations of structural and predicted features and also possible interactions among structural features. Therefore, in the subsequent analysis, we focus mainly on the prominent folding and binding constraints that are likely to suffer the least from these potential uncertainties.

### Folding and binding act as strong constraints

Protein folding and ligand binding are known to act as spandrels underlying mutational fitness effects [47,48]. Here we demonstrate that the two factors act as strong constraints on fitness of GmR mutants. In order to further understand the influence of these two coupled constraints, we created four subsets of mutants with unique combinations of protein folding and ligand binding states: (1) both proper (i.e. non-compromised) folding and binding (FB), (2) compromised folding and proper binding (cFB), (3) proper folding and compromised binding (FcB) and (4) both compromised folding and binding (cFcB). Here, F and B denote proper folding (low ΔΔG) and proper binding (high distance from active site) respectively, whereas cF and cB denote compromised folding (high ΔΔG) and compromised binding (low distance from active site) respectively. Median values of ΔΔG and distance from active site for all mutants are used as cut-offs in assigning the subsets. Additionally, in order to reduce influence of the uncertainties involved in the estimations of the structural features, mutants whose values lie within 10 percentiles around the median cut-off were excluded.

**Fig 4:**
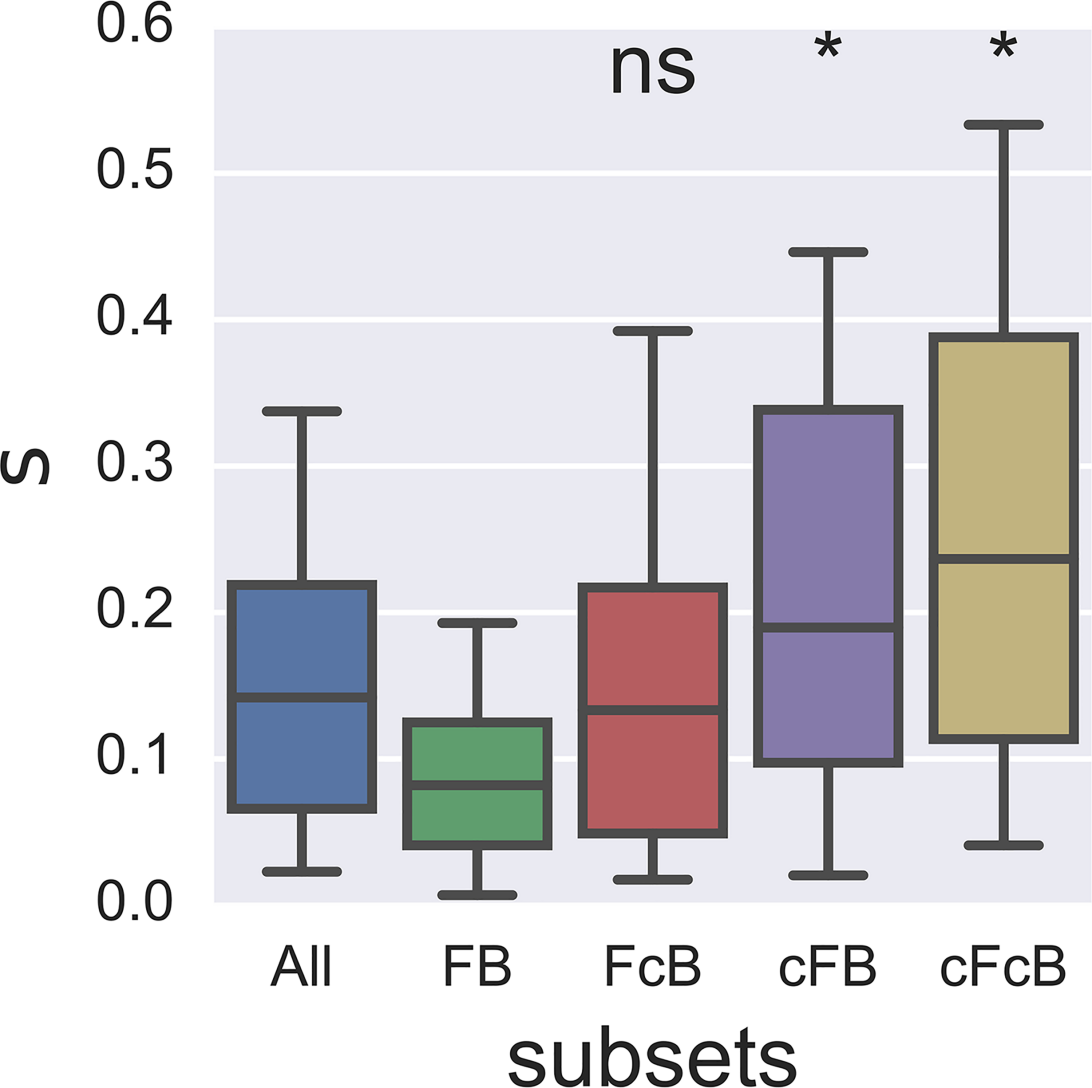
Folding and binding act as strong constraints. Subset-wise mean viability selection coefficients (s) (median ± 50 & 95% of the distribution) across environments. Significance of differences between subset FB and rest of the subsets (FcB, cFB and cFcB) were determined by one-sided Mann-Whitney U tests where mean viability selection in each environment was considered as one paired observation for the four groups (*, P < 0.05, ns, non-significant).

In order to understand how environmental sensitivity of folding and binding perturbations affect mutational GEIs, cross-environment correlations of fitness scores were carried out through Bayesian resampling for each of the four mutant subsets separately (S8A Fig, S3 Table and S1 Text). The correlations between 30°C and 37°C were strong and close to unity and did not differ between the four subsets (all P_MCMC_ > 0.2, S8A Fig), recapitulating the similarity in selection pressures across these temperatures. However, the correlations between 42°C and the other two test temperatures were significantly lower for the subsets of mutants with compromised folding or binding (cFB and FcB compared to FB; all P_MCMC_ < 0.001, S8B & S8C Fig), again pinpointing folding and binding constraints as central in determining environmental specificity of mutational fitness effects. Next, subset wise mean viability selection coefficients were determined for all the environments (S4 Table). Across all the environments, a pronounced trend of increased mean viability selection with compromised folding and binding is evident: ‘FB < FcB < cFB < cFcB’. Folding constraints in particular impose the largest and statistically significant (P<0.05) increase in mean viability selection coefficients; implying that it may act as a stronger constraint among the two (Fig 4).

**Fig 5:**
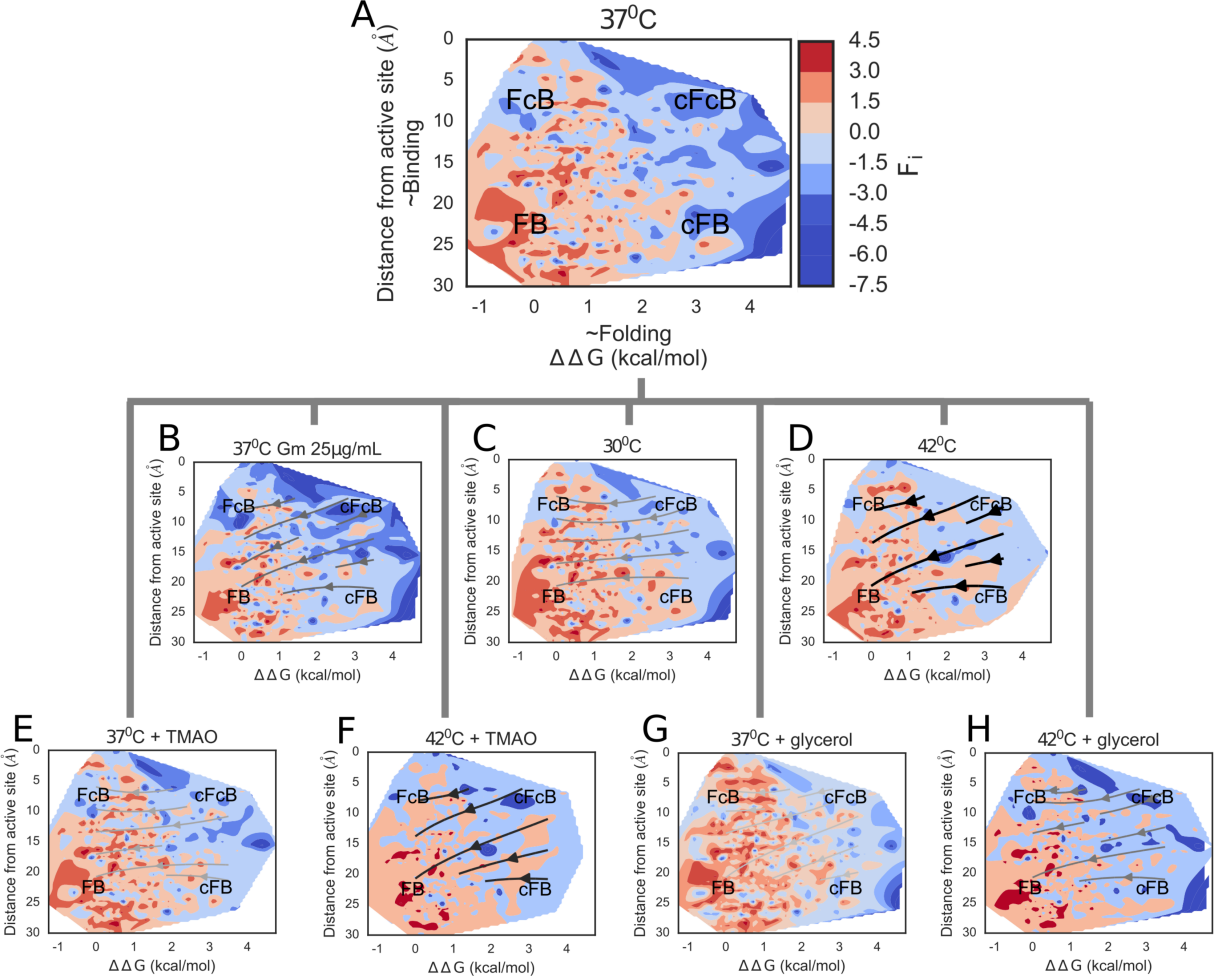
Unique environmental reshaping of fitness landscapes. Landscapes are plotted in the form of contour plots, outlined by folding (ΔΔG (kcal/mol)) and binding (distance from active site) components with colors delimiting the fitness scores (F_i_) of surviving mutants. Contour surfaces are generated by nearest neighbor interpolation. Regimes at the corners of the fitness landscapes represent subsets of mutants i.e. FB, cFB, FcB and cFcB. Colors of all contour plots are scaled according to the colorbar associated with panel A. Streamlines on plots B-H are directed towards fitness maxima in each case: from high *s* (i.e. highly deleterious) mutations to low *s* (~ neutral) mutations. The intensity of selection (magnitude of *s*) in each environment is indicated by the darkness of the streamlines. Streamlines on landscapes with high s are colored in darker shades.

Further, utilizing the predictability of folding and binding constraints in determining mutational fitness, we visualized the environmental effects in the form of low-dimensional fitness landscapes (Fig 5). Outlined by the constraints, regimes at the corners of the landscapes represent the four subsets of mutants i.e. FB, FcB, cFB and cFcB. The fitness landscape in the reference environment seems to be shaped by folding constraint, producing a pronounced fitness cliff at ΔΔG~2 kcal/mol separating high and low fitness mutants (Fig 5A). In contrast, at the stringent Gm concentration, mutations close to the active site (i.e. cB subsets) show a prominent decrease in fitness (Fig 5B), corroborating the observed dosage dependent effects reported above. Indeed, the imposed higher load of Gm seems to generate an additional pronounced fitness cliff along the binding axis–at a ~15Å distance from the active site.

Among the physical environments, the fitness landscape at the low temperature condition (Fig 5C) show no clear difference from that of the reference environment; echoing the earlier noted weak environmental effect on selection. Contrastingly, elevated temperature conditions show reduced survival of mutants, especially at cFB and cFcB regimes (Fig 5D), signifying a strong influence of folding constraints. Among chemical environments, the mutational robustness conferred by TMAO and glycerol at 37°C is evident from the close similarity of these fitness landscapes and that of the reference environment (Fig 5E and 5G). At 42°C though, partial assistance is evident in FB subset (Fig 5F and 5H). Notably, across all the fitness landscapes, the common existence of fitness cliffs along the folding axis suggests that folding constraint is universally strong among all the environments. This in turn also explains the conformity between the anticipated alteration of protein folding by each environment and corresponding selection pressures. Overall, visualizing the complex environmental effects on fitness of GmR mutants through the perspective of molecular constraints reveals a shaping of mutational fates that is closely dependent on the inherent strengths of the molecular constraints.

## Discussion

Large-scale elucidations of genotype-by-environment interactions (GEI) and the environmental specificity of mutational fitness effects enabled by high throughput mutational scanning [22] have opened up new possibilities to comprehensively assess fundamental questions in molecular evolution. Here, we linked environment-specific competitive fitness of mutants to the underlying molecular basis of GEI, by deep mutational scanning of the antibiotic resistant gene GmR.

Upon monitoring empirical fitness of a library of single site mutants of the gene, under sets of physical and chemical environments, we characterized corresponding selection pressures. In line with earlier findings [16,26,49], we demonstrate that the environment can significantly change selection and the fitness consequences of de novo mutations (Fig 2). Among physical environments, elevated temperature (42°C) exerts strong selection against non-synonymous mutations, underscoring overall temperature sensitivity [31] upon protein misfolding [42]. Low temperature (30°C), on the other hand, imposes comparatively weaker selection, conforming to known non-deleterious effects on protein folding at low temperature [50]. Among chemical environments, chemical chaperones too exert weaker selection, while when applied in combination with high temperature, they even alleviate selection pressure imposed by high temperature; underscoring earlier results identifying mutational buffering properties [34]. The alleviation of deleterious effects of elevated temperature by chemical chaperones also indicates a partial additivity and therefore a degree of predictability in the action of complex environments. The reason for this degree of predictability can likely be attributed to the heterologous expression of GmR that made mutant fitness directly dependent on the properties of a single gene. This is in contrast with a previous study in which GEIs of an endogenous gene–Hsp90 were found to be largely unpredictable [16]. Participation of Hsp90 in dense signaling networks of stress response pathways [51] may have potentially obscured the predictability in that case. For example, a candidate mutation that rendered Hsp90 inactive at high temperature while maintaining activity at high salinity can be equally well explained by two alternative hypotheses: either the Hsp90 mutant misfolded specifically at high temperature, or the temperature-specific signaling through Hsp90 was abrogated. By contrast, our work pinpoints the former factor as the main contributor mutational effects and illustrates the utility of the used experimental system for the study of evolution of structure and function in the context of environmental change. The next step will be to integrate protein-protein interactions and signaling networks to define environmental effects on higher levels of GEIs.

The correlative analysis (Fig 3) identified protein stability perturbations (ΔΔG) and perturbations of ligand binding (distance from active site) as strong molecular constraints on fitness, and hence determinants of environment-specific mutational fitness effects. This finding is in line with the proposed spandrel-like properties of these two constraints [47,48]. Our measures of fitness scores were highly repeatable (S2 Fig). Assuming the same accuracy in estimating the structural features of the mutations, the generally weak (r_s_<0.5) correlations between these two estimates indicate that fitness scores of only a fraction of mutants were explainable by any individual structural feature. This may suggest that some of these molecular features (e.g. folding and binding) have interactive effects on fitness, necessitating accounting for this dependence to better predict mutants’ fitness. Additionally, potential non-monotonic relationships would also contribute in weakening the strengths of the correlations [27].

In this study, we extended the results of previous studies [7,39,52] to understand how the effective contribution to molecular constraints change in different environments. The central role of both constraints in shaping fitness effects in different environments was evident from the subset-wise mean viability selection coefficients, where environmental effects are more pronounced in subsets of mutants with compromised folding and binding (Fig 4). Among the two constraints, however, folding seem to introduce a prominent limiting fitness cliff (at ΔΔG = ~2 kcal/mol on the folding axis of Fig 5) across most of the environments. However, the relative strengths of the constraints were context dependent. For example, we observed that the binding constraint emerges to be stronger as the antibiotic concentration is elevated. These results conform to other studies (e.g. [52]) showing that biophysical constraints dictate mutational tolerance. Overall, our findings thus suggest that GEI associated with de novo mutations can be understood in terms of environmental alteration of protein folding and binding constraints, which is in alignment with their central role in molecular evolution [18,19].

Collectively, from a simple experimental system consisting of a conditionally essential gene, we identify that environment-specific mutational fitness effects are dependent on the relative strengths of underlying molecular constraints. The heterologous gene expression produced relatively predictable GEIs that opened up possibilities to contextualize fairly complex GEIs of endogeneous genes, as well as to forecast molecular evolution in complex environments, premises that only recently would have seemed a daunting and perhaps unrealistic task. A mechanistic understanding of GEIs is arguably one of the most important challenges when predicting evolution of complex traits [1] and innovations [53]. Information such as we present here may considerably advance our understanding of the molecular underpinnings of the genotype-phenotype map and how the materialization of molecular constraints shape phenotypic evolution in complex environments [49,54]. Moreover, including knowledge about how the environment may induce phenotypic variability, or alter the fitness consequences of allelic variants, can potentially increase the robustness and accuracy of predictions of phenotypic outcomes of genomic variants [29,30]. In the future, the comprehensive approach utilized here to elucidate environment-specific fitness landscapes can be extended to monitor intragenic and intergenic epistasis.

## Materials & methods

### Minimal inhibitory concentration (MIC) assays

The primary culture was prepared by inoculating (1% v/v) *E*. *coli* (K-12) in culture media (Luria-Bertani (LB) broth (HiMedia) containing 100μg/mL, ampicilin (Sigma) and 0.1% Arabinose (Sigma)) and incubating at 37°C for 18 hrs. The primary culture was inoculated at OD_600_ of 0.025 in culture media containing a range of Gm (Sigma) concentrations from 6.25 to 400 μg/mL with 2 fold increase at each increment (in 96-well storage plates). The assay plates were incubated at 37°C for 18 hrs before measuring growth (OD_600_) in Tecan microwell plate reader.

### Growth assays

*E*. *coli* (K-12) harboring pBAD-GmR is grown in culture media (LB media containing 100μg/mL and ampicilin 0.1% Arabinose) for ~18 hr. The primary culture was used as an inoculum (~0.01 OD) for the growth assays. Growth assays in different environments were carried out using Bioscreen C kinetic growth reader. The growth parameters were obtained by fitting absorbance data to a five parameter Logistic equation.

### Co-culture bulk competition assay

An SSM library of GmR was constructed by PCR based site directed mutagenesis, using primers with degenerate codons (NNK). For detailed information regarding the mutagenesis, please refer to Supporting methods described in Bandyopadhyay et al. [34]. For co-culture bulk competition assays, the mutation library cloned in pBAD vector was transformed into *E*. *coli* (K-12). Primary culture was prepared by inoculating pool of SSM library (1% v/v) in culture media (LB media containing 100μg/mL ampicilin and 0.1% Arabinose) at 37°C for 18 hrs. A competition was carried out at the secondary culture where primary culture in inoculated at OD_600_ of 0.025 and incubated for 18 hrs. Physical environmental conditions were created by carrying out the bulk competitions at 30°C (low temperature) or 42°C (elevated temperature). Chemical environmental conditions were created by supplementing either TMAO (250mM) or glycerol (250mM) in the culture media of competition assay. Biological replicates were made by carrying out independent co-culture bulk competitions of the mutant libraries. For measuring fitness of mutants in a particular environmental condition, bulk competition under Gm selection (selected pool) (as shown in Fig 1A) was carried out. An independent bulk competition was carried out at 37°C in the absence of Gm (unselected pool) which serves as a reference for calculating preferential enrichments.

### Deep sequencing

At the end of bulk competition assays, cells are pelleted and plasmid is purified. Amplicons were generated by a short PCR (initial denaturation: 95°C for 3 min, denaturation: 95°C for 1 min, annealing: 60°C for 15 sec, extension: 72°C for 1 min, final extension: 72°C for 10 min) using high fidelity KAPA HiFi DNA polymerase (cat. no. KK2601). High template concentration (1 ng/μl) and 20 cycles were used to reduce potential PCR bias. Multiplexing was carried out using flanking barcoded primers (4 forward, 4 reverse, sequences in S5 Table). Amplicons of barcoded samples were grouped in equimolar concentration and gel purified. A dual index library for each such set was prepared using Truseq PCR-free DNA HT kit (Illumina Inc. Cat no. F-121-3003) and sequenced using paired end (300 X 2) chemistry on Illumina Miseq platform. Raw sequencing data is available at Sequence Read Archive (SRA) as a BioProject: PRJNA384918.

### Estimation of fitness scores from deep sequencing data

Analysis of sequencing data was carried out by using *dms2dfe* [55]—a comprehensive analysis pipeline exclusively designed for analysis of deep mutational scanning data. Through *dms2dfe* workflow, output files from the sequencer (.fastq) were demultiplexed using *ana0_fastq2dplx* module of *dms2dfe*. Average read depth of each demultiplexed sample was ~1X10^5^. Next, though *dms2dfe*'s modules namely *ana0_fastq2sbam*, sequence alignment was carried out using Bowtie2 [56], followed by variant calling through *ana1_sam2mutmat* module which utilizes *pysam* libraries [57]. A variant is called only if average Q-score of the read and that of the mutated codon is more than 30. Additionally a cut off of 3 reads per variants is used to filter out anomalous low counts. As a result a codon level mutation matrix of counts of mutations is generated. Codon level mutation matrix is then translated to amino acid level (based on the codon usage bias of the *E*. *coli*). For each experimental condition, counts of ~2000 individual mutants were quantified (S1 Data). Raw sequencing data is available at Sequence Read Archive (SRA) as a BioProject: PRJNA384918.

Through *ana2_mutmat2fit* module of *dms2dfe*, counts of mutants are first normalized by the depth of sequencing at each position of the gene. Then preferential enrichments which are log (base 2) fold change of counts of the mutants in pool selected in presence of Gm against unselected (0 μg/mL Gm) reference pool were estimated. Here, preferential enrichment of a mutant serves as a proxy for its relative fitness and hence we simply refer it as ‘fitness’ (S1 Data).

Upper and lower thresholds for statistically neutral fitness effects were defined by adopting a strategy from a similar previous study [41]. As shown in S4 Fig, the thresholds were obtained as mean ± two SD from a distribution of F_i_ obtained from unselected condition.

### Comparison of environment specific fitness effects

We analyzed the survival of all 2104 non-synonymous mutants (S1 Data), in each of the seven environments, as a binomial response (presence/absence) in logistic regression using Bayesian Markov Chain Monte Carlo simulations in the MCMCglmm package [58] for R [59]. Temperature (30, 37 or 42°C), treatment (reference, glycerol or TMAO) and their interactions were included as fixed effects. We ran the model with residual variance fixed to 1 and a flat prior on the probability scale for the fixed effects, recommended when the number of observations in some cells are low [60] (as for mutant absence in some environments; S1 Data) and the data show near complete separation [61]. The model ran for 2000000 simulations preceded by 200000 burn-in simulations that were discarded. We stored every 2000^th^ simulation, resulting in 1000 uncorrelated posterior estimates of mean mutant survival in each environment. As 30°C was only applied using reference media, we analyzed differences between 30 and 37°C separately. Similarly, as the Gm25 treatment only was applied across the different media at 37°C, the comparison between Gm 25μg/mL and 12.5μg/mL was analyzed in a separate model.

To formally estimate the influence of environment on the magnitude of fitness effects and the strength of selection on de novo mutation we compared the viability of the non-synonymous mutants to that of the 157 synonymous mutants. Hence, mean viability selection coefficients (**s**) against the non-synonymous mutations in each environment (*i*) was estimated as:

$$s=1–\left[v_{i}^{non}/v_{i}^{syn}\right]$$

where *v*_*i*_^non^ and *v*_*i*_^syn^ is the mean survival of the non-synonymous and synonymous mutants, respectively, in environment *i*. We utilized the 1000 stored Bayesian posterior estimates of mean viability of the non-synonymous mutants (*v*_*i*_^non^) (S6 Table), and then generated 1000 matching estimates of *v*_*i*_^syn^ by applying the equivalent Bayesian analysis described above to the synonymous mutant data. We then used these two posterior distributions to calculate *s* per environment and tested if the generated posterior distributions of *s* differed significantly across environments at an alpha level of 0.05.

In addition to these selection coefficients, we provide three relative measures of fitness effects for comparison with reference environment.

1. Relative change in average fitness scores (ΔF) was calculated as the difference between average fitness of a given environment and reference environment.
2. Mutational robustness score (ρ) was quantified as the rank correlation coefficient between fitness scores of a given environment and the reference environment.
3. Ratios of the number of mutants undergoing positive (n_pos_) and negative (n_neg_) effects compared to the reference environment (i.e. n_pos_ /n_neg_) were determined. To achieve this, statistical thresholds were assigned to demarcate inherent noise within replicates of both test and reference environment conditions. If μ_test_ and μ_reference_ are means and σ_test_ and σ_reference_ are standard deviations of fitness changes ‘within replicates’ for test and reference environments, the statistical thresholds for noise was determined to be equal to $\frac{{µ}_{test}+{µ}_{control}}{2}\pm 2*\sqrt{\frac{\sigma_{test}^{2}+\sigma_{control}^{2}}{2}}$. Mutants that have a fitness change ‘between environments’ which is greater than the threshold are considered to undergo ‘positive’ effects. Likewise, mutants that have fitness change across environments which is lesser than the threshold, are considered to undergo ‘negative’ effects.

Values of all four parameters for all the environments are included in S1 Table.

### Structural features of GmR

Mutant stability perturbations (ΔG) are predicted by PoPMusic [44] server. Evolutionary rate per site (conservation score) is acquired from ConSurf (7) server. MSMS libraries [45] were used for calculations of residue depth from surface of protein. Distances between atoms of GmR are measured using various modules of Biopython package [62]. Distances of residues (mutation sites) from active site residue D147 are estimated. Here, minimum distance between the atoms of the D147 and C-alpha atom of a given residue is used to ensure maximum sensitivity. Physico-chemical properties of the amino acids such as logP and pI were retrieved from PubChem [63] and ChemAxon (http://www.chemaxon.com). Structural features of mutations used in the study are included in S2 Data.

## Supporting information

S1 Fig Optimizing Gm concentration for co-culture bulk competition assays. (A) Growth kinetics of wild type GmR (pBAD-GmR) under a range of dosages of Gentamicin are shown. Maximum asymptote values were obtained by fitting growth curves to five parameter logistic equation. (B) Extent of growth of E. coli K-12 with (pBAD-GmR) and without (Untransformed) wild type GmR obtained by minimal inhibitory concentration (MIC) assay.
(TIF)

S2 Fig Reproducibility among biological replicates. Correlations among counts of mutants (log-scaled) from independent biological replicates are shown. r is the Pearson’s correlation coefficient.
(TIF)

S3 Fig Mutation map under reference environment i.e. 37°C at 12.5 μg/mL Gm. F_i_ is fitness level of individual mutant. Each row in the heatmap represents mutated amino acid while each column represents reference amino acid. The values of heatmap are scaled by the fitness score (F_i_). In the panel representing secondary structures, H denotes Helix, E denotes beta-sheets, T denotes turns and S denotes bends. Mutated amino acids in rows are grouped by similarities. The groups of amino acids and corresponding colors are as follows. Non polar: red, neutral: green, neutral polar: blue, positively charged: orange, negatively charged: magenta, aromatic: cyan. Mutations for which data is not available are denoted by ‘⊗’ symbol. Synonymous mutations are marked by ‘+’ symbol.
(TIF)

S4 Fig Estimation of cut-offs for classification of mutants as enriched or depleted. These are determined from a distribution of preferential enrichments between replicates of unselected pools (0μg/mL). μ and σ are the mean and standard deviations of the distribution respectively. F_i_ is fitness score.
(TIF)

S5 Fig Distributions of fitness scores of synonymous mutations across different test environments. F_i_ is fitness score. μ is mean and σ is standard deviation.
(TIF)

S6 Fig Correlation between conservation scores and fitness of mutants under reference environment i.e. 37°C at 12.5 μg/mL Gm. F_i_ is fitness score of individual mutant. Hex colors are scaled according to distance of the mutation site from the active site of the protein.
(TIF)

S7 Fig Comparison of DFEs obtained for the treatment of chemical chaperones at 25μg/mL Gm with 37°C 25μg/mL Gm. F_i_ denotes fitness score, *s* denotes mean viability selection coefficient. Significant differences between the viability selection coefficient in a specific test environment compared to the control environment (37°C, 12.5μg/mL) was evaluated by Bayesian MCMC resampling (***, P < 0.001, **, P < 0.01, See Materials and Methods). ΔF is relative change in average fitness. ρ is a mutational robustness score. Distributions are fitted by kernel density estimation. Boxplots show median ± 50 & 95% of the distributions.
(TIF)

S8 Fig Environmental specificity of folding and binding constraints. (A,B and C) Bayesian posterior estimates (median ± 50 & 95% of the distribution) of mutational correlations across the three temperatures for the four subsets of mutants based on their binding (B/cB) and folding (F/cF) constraints. Bayesian posterior estimates (and 95% credible intervals) of correlations are included in S3 Table.
(TIF)

S1 TableMetrics of the distributions of fitness under different environmental conditions.n^non^, n^syn^: total non-synonymous and synonymous mutants survived respectively. *v*^*non*^, *v*^*syn*^: fraction of non-synonymous and synonymous mutants survived respectively
$$(v=\frac{n}{total\;number\;of\;mutants\;in\;the\;library}).$$
total number of non-synonymous mutants in the library = 2104total number of synonymous mutants in the library = 157*s*: mean viability selection coefficient.n_pos_/n_neg_: ratio of counts of total number of mutants undergoing positive and negative effects respectively with respect to reference environment i.e. 37°C.ΔF: Difference between average fitness of mutants in the given environment and reference environment i.e. 37°C.ρ: Rank correlation coefficients between fitness scores of a given environment and reference environment.(XLSX)

S2 Table Correlations between fitness scores of mutants and molecular features. ****: P < 0.0001, ***: P < 0. 001, **: P < 0.01, *: P < 0.05, ns: non-significant.
(XLSX)

S3 Table Bayesian posterior estimates (and 95% credible intervals) of mutational correlations across the three temperatures, for four subsets of mutants. (XLSX)

S4 Table Subset-wise mean viability selection coefficients. (XLSX)

S5 Table Barcoded primers used to multiplex amplicons of GmR. 7 nucleotide long barcodes sequences are highlighted in bold.
(XLSX)

S6 Table Comparison of posterior distributions to assess significant differences in fitness effects of non-synonymous mutations between environments. Posterior means and Bayesian P-values (pMCMC) are given as marginal contrasts where fitness effects at 37°C and Gm 12.5 μg/mL is taken as the model intercept to which all other main effects are compared. Significant higher order interactions (e.g. 42°C + TMAO) indicate that the mutational fitness effects are significantly different in the test environment than what would have been predicted given the mutational fitness effects observed in each of the environments (i.e. 42°C and TMAO) in isolation.
(XLSX)

S1 Text Supporting methods. (DOCX)

S1 Data Fitness scores of mutations in different environments. (XLSX)

S2 Data Structural features of mutations. (XLSX)
