# Supplementary figures and images for: Differential strengths of molecular determinants guide environment specific mutational fates
Source: PLoS Genet. 2018 May 29;14(5):e1007419. doi: 10.1371/journal.pgen.1007419 (PMC5993328; doi:10.1371/journal.pgen.1007419)

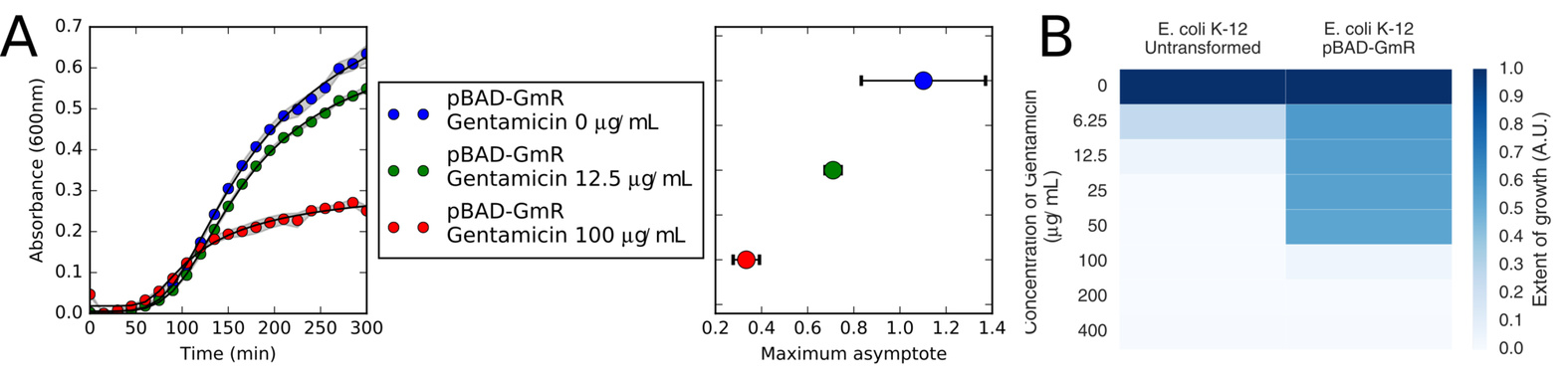

Supplement: S1 Fig — (A) Growth kinetics of wild type GmR (pBAD-GmR) under a range of dosages of Gentamicin are shown. Maximum asymptote values were obtained by fitting growth curves to five parameter logistic equation. (B) Extent of growth of E. coli K-12 with (pBAD-GmR) and without (Untransformed) wild type GmR obtained by minimal inhibitory concentration (MIC) assay. (TIF) [file pgen.1007419.s001.tif]

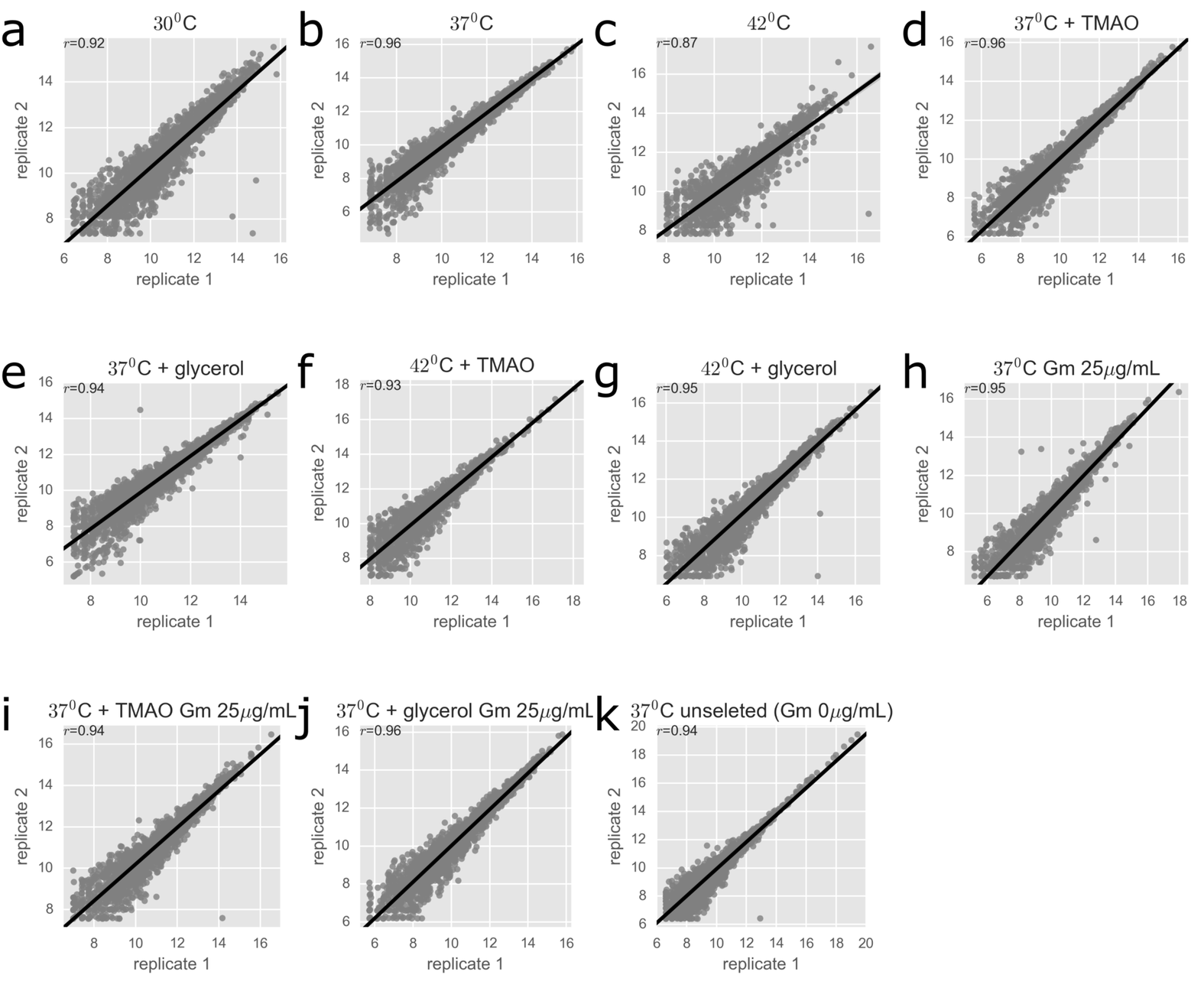

Supplement: S2 Fig — Correlations among counts of mutants (log-scaled) from independent biological replicates are shown. r is the Pearson’s correlation coefficient. (TIF) [file pgen.1007419.s002.tif]

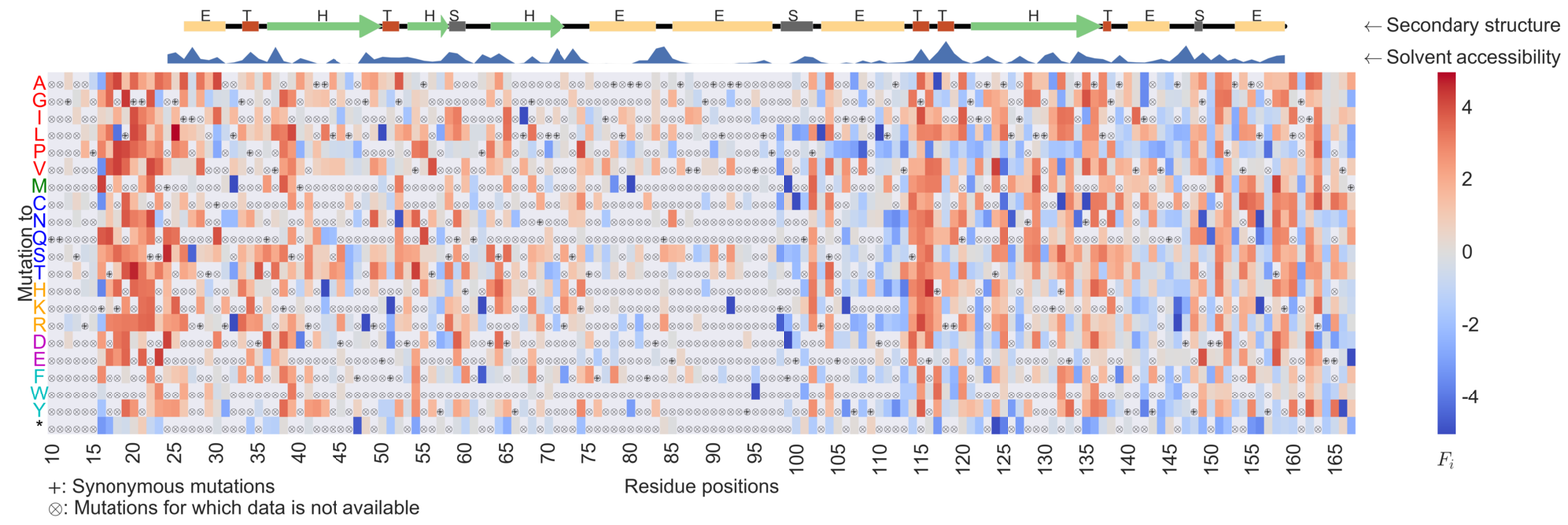

Supplement: S3 Fig — Fi is fitness level of individual mutant. Each row in the heatmap represents mutated amino acid while each column represents reference amino acid. The values of heatmap are scaled by the fitness score (Fi). In the panel representing secondary structures, H denotes Helix, E denotes beta-sheets, T denotes turns and S denotes bends. Mutated amino acids in rows are grouped by similarities. The groups of amino acids and corresponding colors are as follows. Non polar: red, neutral: green, neutral polar: blue, positively charged: orange, negatively charged: magenta, aromatic: cyan. Mutations for which data is not available are denoted by ‘⊗’ symbol. Synonymous mutations are marked by ‘+’ symbol. (TIF) [file pgen.1007419.s003.tif]

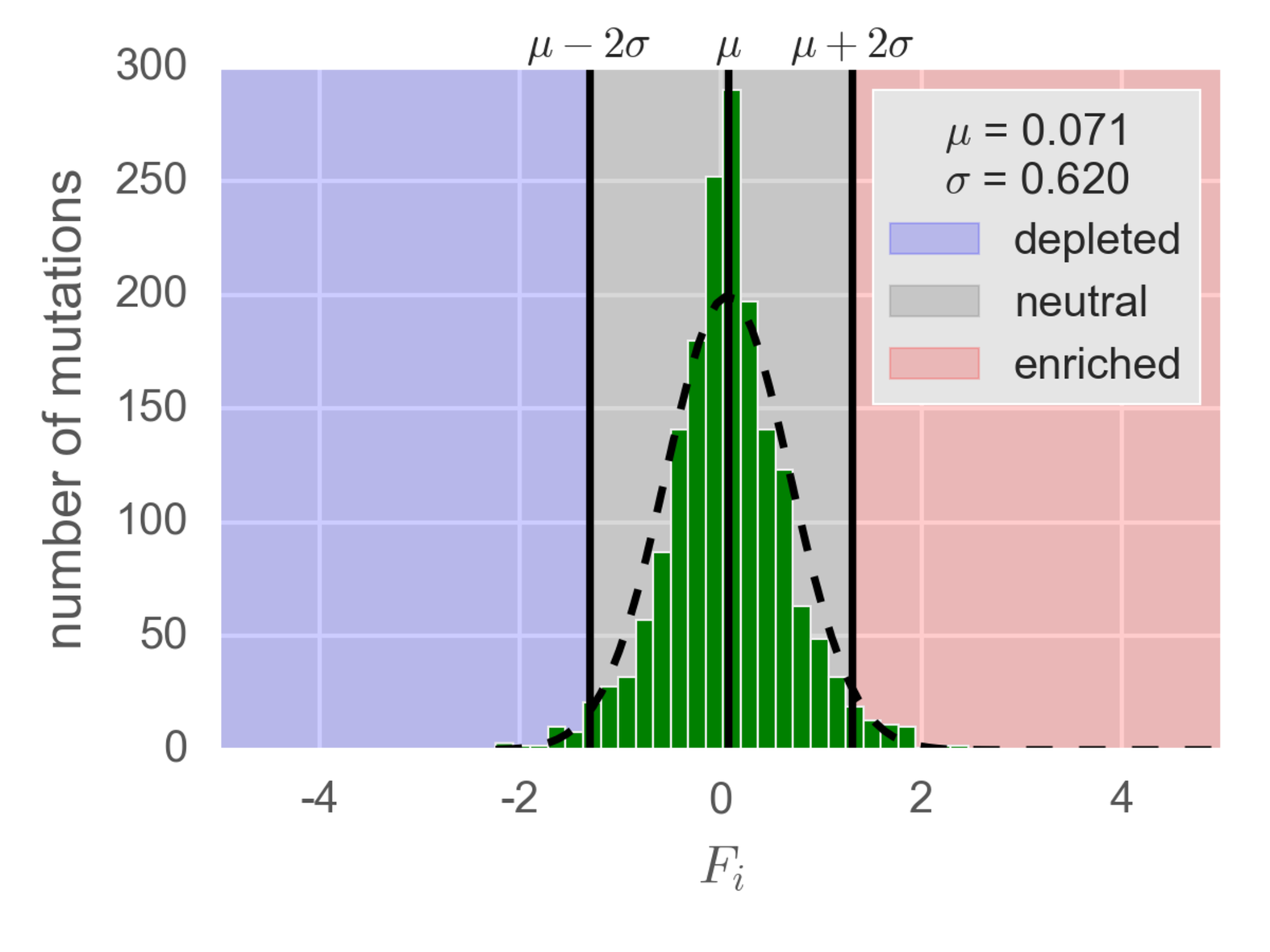

Supplement: S4 Fig — These are determined from a distribution of preferential enrichments between replicates of unselected pools (0μg/mL). μ and σ are the mean and standard deviations of the distribution respectively. Fi is fitness score. (TIF) [file pgen.1007419.s004.tif]

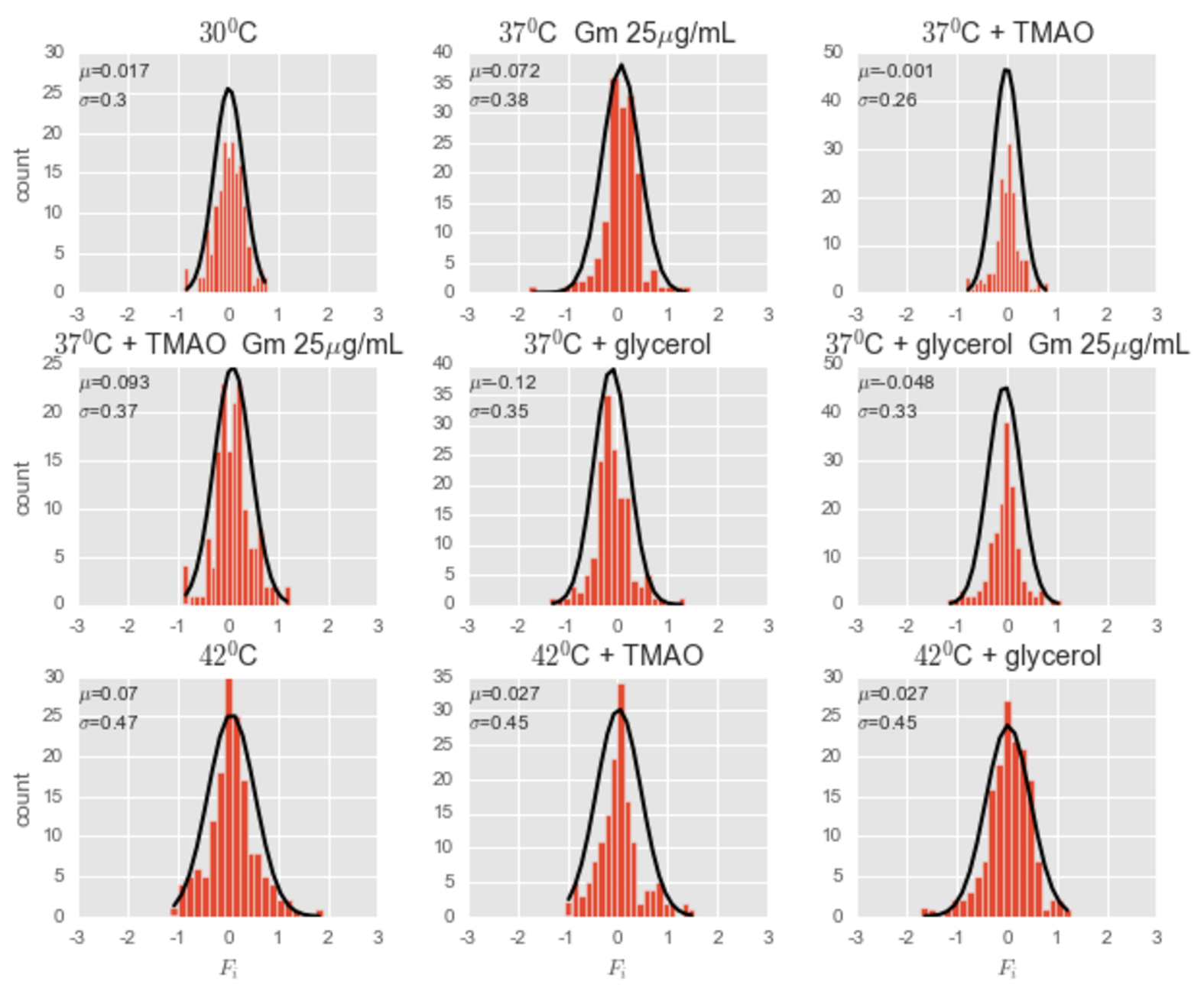

Supplement: S5 Fig — Fi is fitness score. μ is mean and σ is standard deviation. (TIF) [file pgen.1007419.s005.tif]

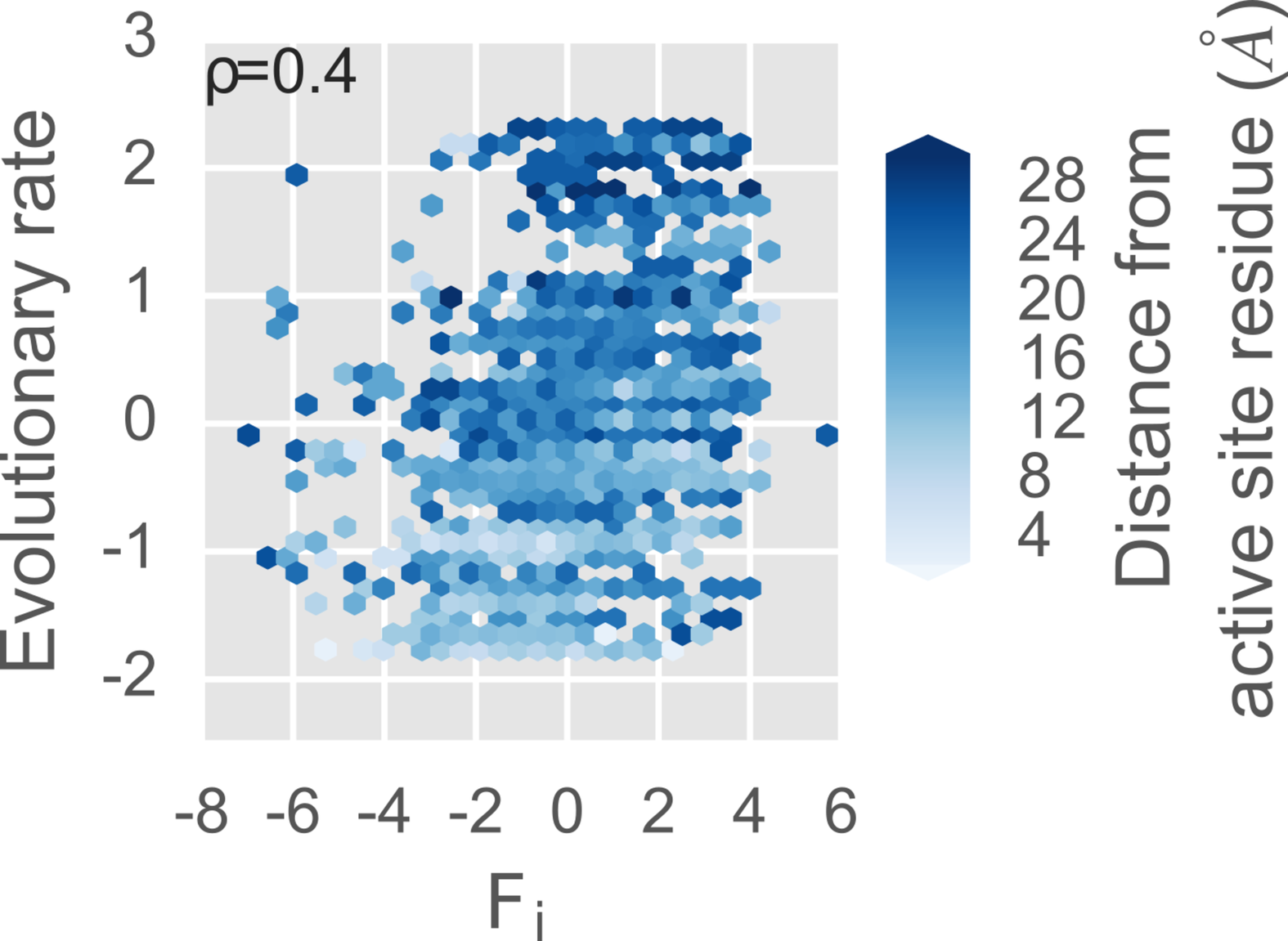

Supplement: S6 Fig — Fi is fitness score of individual mutant. Hex colors are scaled according to distance of the mutation site from the active site of the protein. (TIF) [file pgen.1007419.s006.tif]

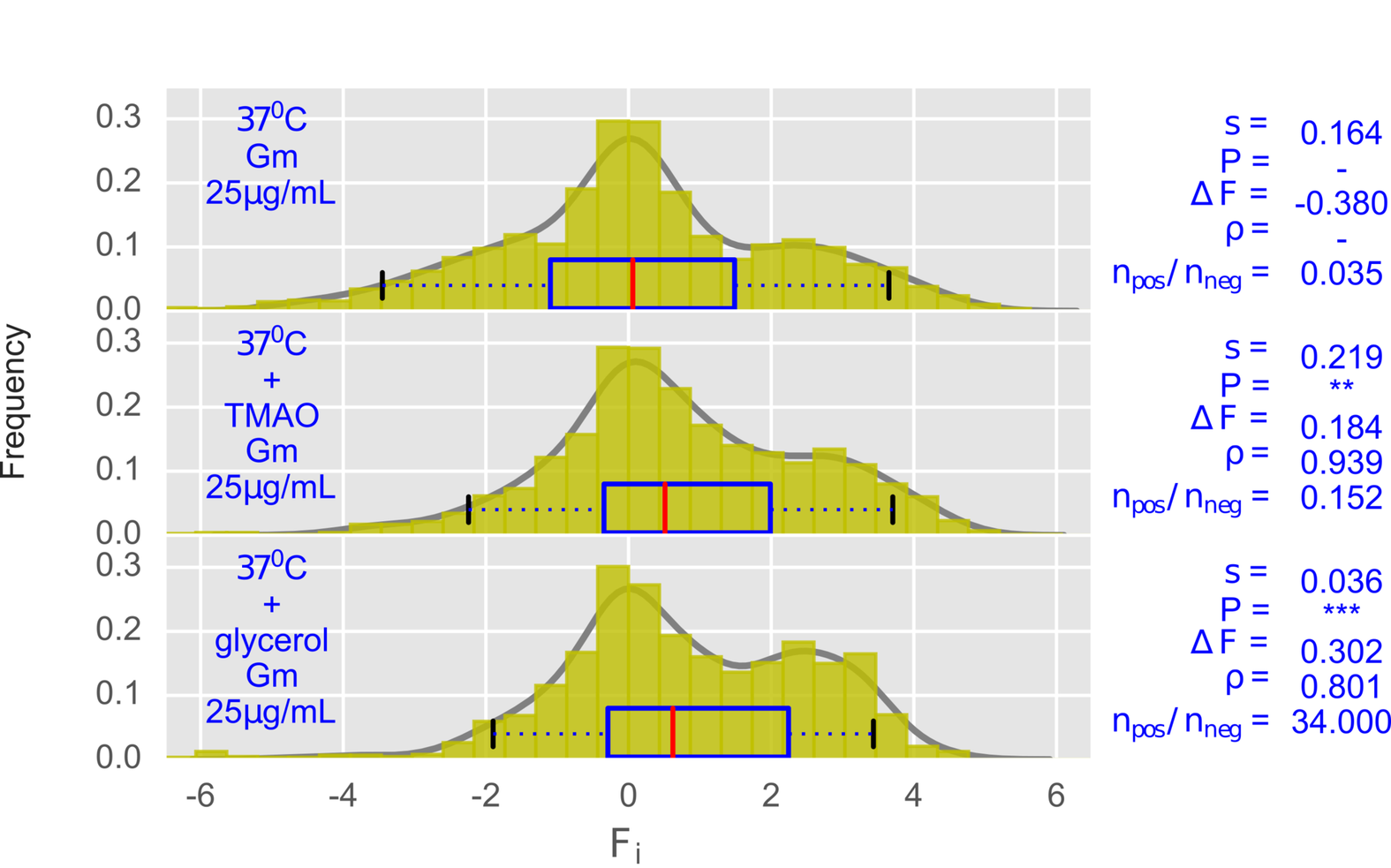

Supplement: S7 Fig — Fi denotes fitness score, s denotes mean viability selection coefficient. Significant differences between the viability selection coefficient in a specific test environment compared to the control environment (37°C, 12.5μg/mL) was evaluated by Bayesian MCMC resampling (***, P < 0.001, **, P < 0.01, See Materials and Methods). ΔF is relative change in average fitness. ρ is a mutational robustness score. Distributions are fitted by kernel density estimation. Boxplots show median ± 50 & 95% of the distributions. (TIF) [file pgen.1007419.s007.tif]

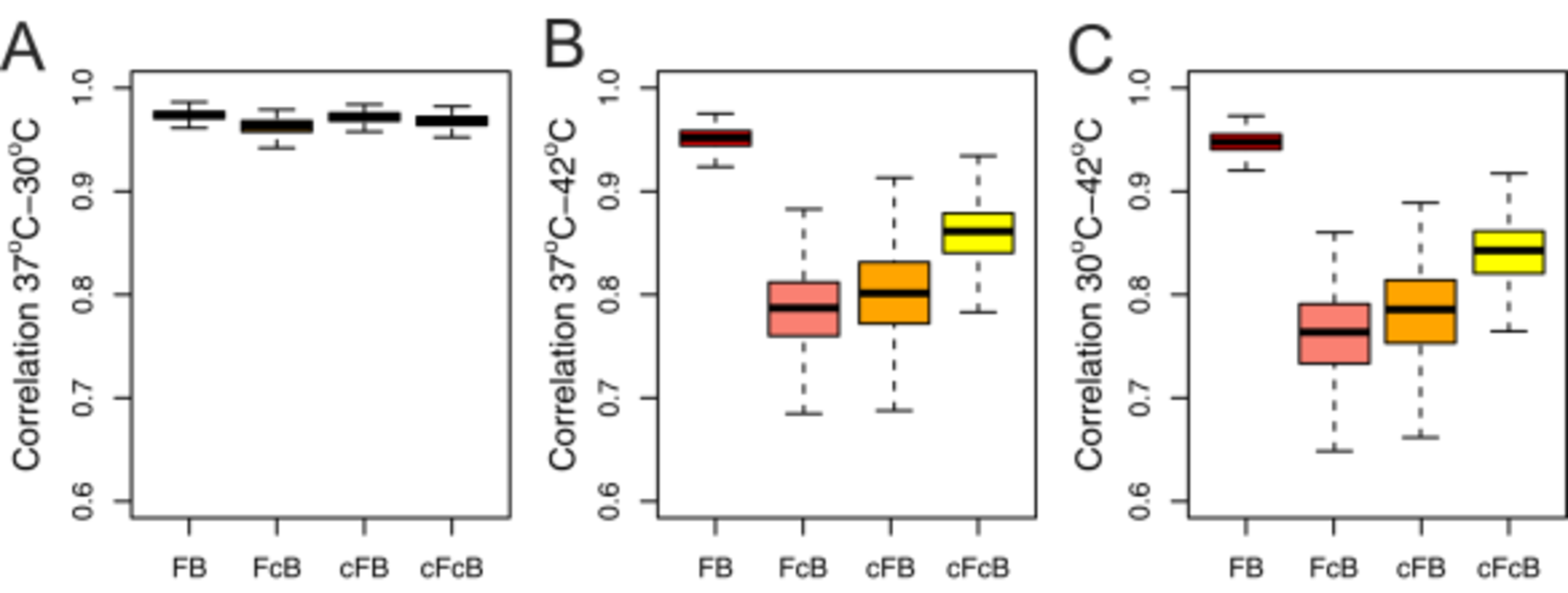

Supplement: S8 Fig — (A,B and C) Bayesian posterior estimates (median ± 50 & 95% of the distribution) of mutational correlations across the three temperatures for the four subsets of mutants based on their binding (B/cB) and folding (F/cF) constraints. Bayesian posterior estimates (and 95% credible intervals) of correlations are included in S3 Table. (TIF) [file pgen.1007419.s008.tif]
