# Supplementary material for: Differential strengths of molecular determinants guide environment specific mutational fates
Source: PLoS Genet. 2018 May 29;14(5):e1007419. doi: 10.1371/journal.pgen.1007419 (PMC5993328; doi:10.1371/journal.pgen.1007419)
Supplement: S1 Text — (DOCX) [file pgen.1007419.s015.docx]

## Supporting Methods

### Bayesian resampling of cross-environment mutational correlations

We applied Bayesian Markov Chain Monte Carlo resampling using the MCMCglmm package (Hadfield 2010) for R (Team R Core 2013) to estimate correlations between mutational fitness across the three test temperatures on the standard growth medium (i.e. the 30, 37 and 42°C environments), for each of the four classes of binding and folding constraints (S8 Figure A, B and C).

Model specification and prior settings were as recommended by Hadfield (2010). The two biological replicates were used to estimate error (i.e. environmental and not mutational) variance when estimating correlations. We ran 120.00 iterations, (of which the first 20.000 were discarded) and stored every 100^th^ resulting in 1000 stored posterior estimates of variance components and correlations. Below is a full summary of model specification and prior settings, as well as model output for the random effects per the four groups of mutants (i.e. variance components used to estimate correlations) as well as the model’s residual variance and fixed effects.

**# prior:**

prior1 = list(R = list(V = diag(1, 3), nu = 0.002),

G = list(G1 = list(V = diag(1,3), nu = 3), G2 = list(V = diag(1,3), nu = 3), G3 = list(V = diag(1,3), nu = 3), G4 = list(V = diag(1,3), nu = 3)))

**# model specification**

MCMCglmm(fitness ~ group*temp2 + repl ,

random = ~

us(temp2:at.level(group,"FB")):mutids +

us(temp2:at.level(group,"cFB")):mutids +

us(temp2:at.level(group,"FcB")):mutids +

us(temp2:at.level(group,"cFcB")):mutids,

rcov = ~ idh(temp2):units,

family="gaussian", prior = prior1,

nitt=120000, slice=TRUE, burnin=20000, thin=100,

verbose = F, pr=F, data=GEI) -> modGEI

**# model summary**

DIC: 9681.074

#Random effects variance components:

"FB" mutids

post.mean l-95% CI u-95% CI eff.samp

30:at.level(group, "FB"):30:at.level(group, "FB").mutids 2.973 2.449 3.544 1111

37:at.level(group, "FB"):30:at.level(group, "FB").mutids 2.968 2.504 3.583 1100

42:at.level(group, "FB"):30:at.level(group, "FB").mutids 2.856 2.323 3.396 1148

30:at.level(group, "FB"):37:at.level(group, "FB").mutids 2.968 2.504 3.583 1100

37:at.level(group, "FB"):37:at.level(group, "FB").mutids 3.127 2.621 3.739 1028

42:at.level(group, "FB"):37:at.level(group, "FB").mutids 2.940 2.409 3.530 1138

30:at.level(group, "FB"):42:at.level(group, "FB").mutids 2.856 2.323 3.396 1148

37:at.level(group, "FB"):42:at.level(group, "FB").mutids 2.940 2.409 3.530 1138

42:at.level(group, "FB"):42:at.level(group, "FB").mutids 3.061 2.483 3.708 1143

"cFB" mutids

post.mean l-95% CI u-95% CI eff.samp

30:at.level(group, "cFB"):30:at.level(group, "cFB").mutids 3.134 2.440 3.803 37:at.level(group, "cFB"):30:at.level(group, "cFB").mutids 3.261 2.627 4.002

42:at.level(group, "cFB"):30:at.level(group, "cFB").mutids 2.307 1.690 3.005

30:at.level(group, "cFB"):37:at.level(group, "cFB").mutids 3.261 2.627 4.002

37:at.level(group, "cFB"):37:at.level(group, "cFB").mutids 3.667 2.888 4.439

42:at.level(group, "cFB"):37:at.level(group, "cFB").mutids 2.558 1.843 3.309

30:at.level(group, "cFB"):42:at.level(group, "cFB").mutids 2.307 1.690 3.005

37:at.level(group, "cFB"):42:at.level(group, "cFB").mutids 2.558 1.843 3.309

42:at.level(group, "cFB"):42:at.level(group, "cFB").mutids 2.792 1.941 3.594

"FcB" mutids

post.mean l-95% CI u-95% CI eff.samp

30:at.level(group, "FcB"):30:at.level(group, "FcB").mutids 3.332 2.760 3.957

37:at.level(group, "FcB"):30:at.level(group, "FcB").mutids 3.281 2.698 3.811

42:at.level(group, "FcB"):30:at.level(group, "FcB").mutids 2.251 1.786 2.841

30:at.level(group, "FcB"):37:at.level(group, "FcB").mutids 3.281 2.698 3.811

37:at.level(group, "FcB"):37:at.level(group, "FcB").mutids 3.421 2.822 4.020

42:at.level(group, "FcB"):37:at.level(group, "FcB").mutids 2.345 1.832 2.911

30:at.level(group, "FcB"):42:at.level(group, "FcB").mutids 2.251 1.786 2.841

37:at.level(group, "FcB"):42:at.level(group, "FcB").mutids 2.345 1.832 2.911

42:at.level(group, "FcB"):42:at.level(group, "FcB").mutids 2.614 1.961 3.268

"cFcB" mutids

post.mean l-95% CI u-95% CI eff.samp

30:at.level(group, "cFcB"):30:at.level(group, "cFcB").mutids 3.140 2.659 3.696

37:at.level(group, "cFcB"):30:at.level(group, "cFcB").mutids 3.213 2.705 3.749

42:at.level(group, "cFcB"):30:at.level(group, "cFcB").mutids 2.306 1.849 2.773

30:at.level(group, "cFcB"):37:at.level(group, "cFcB").mutids 3.213 2.705 3.749

37:at.level(group, "cFcB"):37:at.level(group, "cFcB").mutids 3.512 2.945 4.088

42:at.level(group, "cFcB"):37:at.level(group, "cFcB").mutids 2.493 1.978 2.984

30:at.level(group, "cFcB"):42:at.level(group, "cFcB").mutids 2.306 1.849 2.773

37:at.level(group, "cFcB"):42:at.level(group, "cFcB").mutids 2.493 1.978 2.984

42:at.level(group, "cFcB"):42:at.level(group, "cFcB").mutids 2.404 1.875 2.997

#Environmental variance (per temperature)

post.mean l-95% CI u-95% CI eff.samp

30.units 0.3567 0.3217 0.3895 1177

37.units 0.2821 0.2566 0.3048 1000

42.units 0.4674 0.4123 0.5352 1000

#Fixed effects

post.mean l-95% CI u-95% CI eff.samp pMCMC

(Intercept) 0.407029 0.129201 0.659369 1000.0 0.006 **

groupcFcB -0.554547 -0.880209 -0.222193 810.0 <0.001 ***

groupFB 0.905132 0.556212 1.225705 1000.0 <0.001 ***

groupFcB 0.301248 -0.014761 0.714162 1000.0 0.100

temp237 -0.205312 -0.332750 -0.089460 1039.0 <0.001 ***

temp242 -0.520038 -0.752486 -0.252574 1000.0 <0.001 ***

repltwo -0.026081 -0.065203 0.011485 806.0 0.180

groupcFcB:temp237 -0.080575 -0.221678 0.082691 801.7 0.304

groupFB:temp237 0.127528 -0.010290 0.279892 1188.1 0.090 .

groupFcB:temp237 -0.034479 -0.182682 0.112730 871.3 0.652

groupcFcB:temp242 0.014497 -0.304178 0.335347 1000.0 0.928

groupFB:temp242 0.125525 -0.178788 0.377033 1000.0 0.396

groupFcB:temp242 -0.334461 -0.684589 -0.008127 1000.0 0.054 .

**References:**

Hadfield JD. MCMC methods for multi-response generalized linear mixed models: the MCMCglmm R package. J Stat Softw. 2010;33: 1–22. doi:10.1002/ana.22635

Team, R. Core. "R: A language and environment for statistical computing." (2013).
